# Codon Optimization, Soluble Expression and Purification of PE_PGRS45 Gene from *Mycobacterium tuberculosis* and Preparation of Its Polyclonal Antibody Protein

**DOI:** 10.4014/jmb.2106.06006

**Published:** 2021-09-01

**Authors:** Tao Xu, Minying Li, Chutong Wang, Meili Yuan, Xianyou Chang, Zhongqing Qian, Baiqing Li, Meiqun Sun, Hongtao Wang

**Affiliations:** 1 Department of Clinical Laboratory, School of Laboratory Medicine, Bengbu Medical College, Bengbu, Anhui 233000, P.R. China; 2 Anhui Province Key Laboratory of Immunology in Chronic Diseases, Anhui Key Laboratory of Infection and Immunity, School of Laboratory Medicine, Bengbu Medical College, Bengbu, Anhui 233000, P.R. China; 3 Department of Histology and Embryology, Bengbu Medical College, Bengbu, Anhui 233000, P.R. China; 4 The Infectious Disease Hospital of Bengbu City, Bengbu, Anhui 233000, P.R. China

**Keywords:** *Mycobacterium tuberculosis*, Rv2615c, PE_PGRS45 protein, codon optimization, soluble expression, polyclonal antibody

## Abstract

Studies have demonstrated that PE_PGRS45 is constitutively expressed under various environmental conditions (such as nutrient depletion, hypoxia, and low pH) of the in vitro growth conditions examined, indicating that PE_PGRS45 protein is critical to the basic functions of *Mycobacterium tuberculosis*. However, there are few reports about the biochemical function and pathogenic mechanism of PE_PGRS45 protein. The fact that this *M. tuberculosis* gene is not easily expressed in *E. coli* may be mainly due to the high content of G+C and the use of unique codons. Fusion tags are indispensable tools used to improve the soluble expression of recombinant proteins and accelerate the characterization of protein structure and function. In the present study, His6, Trx, and His6-MBP were used as fusion tags, but only MBP-PE_PGRS45 was expressed solubly. The purification using His6-MBP tag-specific binding to the Ni column was easy to separate after the tag cleavage. We used the purified PE_PGRS45 to immunize New Zealand rabbits and obtained anti- PE_PGRS45 serum. We found that the titer of polyclonal antibodies against PE_PGR45 was higher than 1:256000. The result shows that purified PE_PGRS45 can induce New Zealand rabbits to produce high-titer antibodies. In conclusion, the recombinant protein PE_PGRS45 was successfully expressed in *E. coli* and specific antiserum was prepared, which will be followed by further evaluation of these specific antigens to develop highly sensitive and specific diagnostic tests for tuberculosis.

## Introduction

*Mycobacterium tuberculosis*, the causative pathogen of tuberculosis (TB), is an extraordinarily successful intracellular pathogen [1]. Low efficacy of the Bacillus Calmette-Guérin (BCG) vaccine and increasing multi-drug-resistant strains have led to the re-emergence of TB as a global health threat [2]. Globally, TB resulted in an estimated 10 million new cases and 1.41 million deaths in 2019, and it was the leading cause of death by a single infectious agent (ranking above HIV/AIDS) [3]. In addition, the COVID-19 pandemic has severely affected TB programs and will likely lead to a significant increase in TB cases and deaths, near the global level of TB mortality of the year 2015 [4].

Pro-Glu (PE) and Pro-Pro-Glu (PPE) proteins are named after shared conserved proline (P) and glutamic acid (E) residues in their N-terminal motifs. PE/PPE families including 99 *pe* genes and 69 *ppe* genes in *M. tuberculosis* H37Rv account for approximately 10% of its coding capacity [5]. Analysis of the *M. tuberculosis* H37Rv genome sequence revealed that the *pe*/*ppe* genes are largely limited to members of the pathogenic genus *Mycobacterium*, such as *M. tuberculosis*, *M. bovis*, *M. ulcerans*, *M. marinum*, *M. kansasii*, *M. africanum*, *M. caprae*, and *M. Canettii*, and have unique roles in the virulence, pathogenesis, and persistence of mycobacteria [6]. PE proteins are divided into three subfamilies: PE-only, PE_PGRS, and PPE_MPTR. The PE_PGRS sub-class of proteins contains 63 members, which contain a polymorphic glycine-rich domain that varies in size, sequence, and repeat copy number [7, 8]. The highly antigenic nature of the PE_PGRS proteins, their exclusive presence in pathogenic mycobacteria, and their role in *M. tuberculosis* immune evasion [9], virulence [10], apoptosis [11], and autophagy [12], indicated that these proteins were directly involved in disease development.

*M. tuberculosis* H37Rv PE_PGRS45, encoded by *Rv2615c*, which is located approximately 2.6 MB distal in the genome, is a paralog of PE_PGRS17 (Rv0978c) and PE_PGRS18 (Rv0980c), and is either the progenitor of these two genes or was duplicated from one of them [13]. Bioinformatics analysis on the PE_PGRS proteins was carried out and strong interactions of only the PE_PGRS45 protein containing the phosphorylated motif (DEVpS/DXXpS) to caspase-3 were observed. This indicates that the conserved DEVS/DXXS motif could have evolved for phosphorylation and subsequent recognition by caspase-3. These findings have important implications in unraveling the role of PE_PGRS45 in mycobacterial infection [14].

However, there are few reports on the biochemical function and the role of PE_PGRS45 in the pathogenic mechanism. In the present study, *M. tuberculosis* strain H37Rv recombinant PE_PGRS45 was expressed and purified, and then used to immunize New Zealand rabbits to obtain anti-PE_PGRS45 protein serum to provide reagents for the future investigation of PE_PGRS 45 and its role in *M. tuberculosis* pathogenesis.

## Materials and Methods

### Bacterial Strains, Plasmids, and Reagents

Plasmids pET-28a, pET-32a, pMAL-c5x, and host strains *E. coli* Top10 and Arctic Express (DE3) were all stored in our laboratory. The restriction enzymes, NcoI, XhoI, NdeI, HindIII, and T4 DNA ligase, were purchased from TaKaRa (Japan). Ni-NTA agarose was purchased from Novagen. PCR purification/plasmid isolation kits were obtained from Tiangen (China). Phanta Max Super-Fidelity DNA Polymerase was purchased from Vazyme Biotech (China); Isopropyl β-D-1-thiogalactopyranoside (IPTG) was from Sigma-Aldrich (USA). Protein Marker was purchased from Thermo Scientific; all other chemicals were of analytical grade.

### Construction of Expression Plasmids

The gene sequence encoding PE_PGRS45 (*Rv2615c*) was obtained from *M. tuberculosis* standard strain H37Rv (GeneID: 888215). Codon optimization of the PE_PGRS45 gene sequence was performed using the preferential codon usage for *E. coli*. The optimized sequence was sent to Zoonbio Company (China) for chemical synthesis. The optimized sequence was sub-cloned into pET28a, pET32a, and pMAL-c5x prokaryotic expression vectors. The following primers were used to subclone PE_PGRS45.

The PCR products of PE_PGRS45, pET-28a and pET-32a were digested with NcoI/XhoI, purified, and ligated with T4 DNA ligase at 16°C for 12 h. The pET-28a-PE_PGRS45 and pET-32a-PE_PGRS45 plasmids were transformed into *E. coli* (Top10), respectively. The PE_PGRS45 gene fragment was inserted into the pMAL-c5x expression vector using the NdeI/HindIII sites, and then two fragments were ligated using T4 DNA ligase, yielding the recombinant plasmid pMAL-c5x-PE_PGRS45. All the obtained recombinant plasmids were confirmed by restriction digestion and DNA sequencing. Finally, the confirmed recombinant plasmids were separately transformed into *E. coli* Arctic Express (DE3) for expression studies.

### Expression of Recombinant Plasmids in *E. coli*

A single positive colony of the recombinant *E. coli* Arctic Express (DE3) was picked into 5 ml lysogeny broth (LB) supplemented with 100 μg/ml ampicillin (pET-32a-PE_PGRS45) or 50 μg/ml kanamycin (pET-28a-PE_PGRS45, pMAL-c5x-PE_PGRS45), and grown overnight at 37°C. The culture was diluted (1:100) with 30 ml of fresh LB until an optical density of 0.6 at 600 nm was reached. Afterward, isopropyl-beta-D-thiogalactopyranoside (IPTG) was added to a final concentration of 0.5 mM, and the culture was allowed to grow for 12 h for induction of the recombinant protein at 37°C and 20°C with 200 rpm shaking.

The resulting cell lysates were collected and washed by centrifugation (8,000 g, 15 min, 4°C) and sonicated in PBS phosphate-buffered saline (PBS). The supernatants (periplasmic space) and precipitates (inclusion bodies) were collected, denatured at 100°C for 5 min, and analyzed by 12% SDS-PAGE followed by Coomassie Brilliant Blue staining of the gel.

### Purification of MBP-PE_PGRS45 Protein

Due to the histidine sequence (6 His-tag) at the N-terminal, expression of MBP-PE_PGRS45 protein that has been added by expression plasmid purification was carried out by Nitrilotriacetic acid (Ni-NTA) agarose resin. At 12 h after induction, the cell pellet was collected by centrifugation at 8,000 g for 30 min at 4°C. Pellets were resuspended in lysis buffer (20 mM Tris-HCl, 150 mM NaCl, 1 mM phenylmethylsulfonyl fluoride (PMSF), pH 8.0), and the cells were lysed by ultrasonic homogenization. The crude supernatant cell extract was filtered through a 0.45 μm filter, and was then loaded onto a Ni-NTA agarose resin which had already been equilibrated with the lysis buffer. After loading, the pass-through was collected at a rate of 0.5 ml/min and washed with 100 ml of washing buffer (20 mM Tris-HCl, 150 mM NaCl, 20 mM imidazole, pH 8.0). Finally, the MBP-PE_PGRS45 protein bound on the Ni-NTA resin was eluted from the column with elution buffer (20 mM Tris-HCl, 150 mM NaCl, 250 mM imidazole, pH 8.0) at a flow rate of 0.5 ml/min.

To remove imidazole, the purified protein solution was transferred to a dialysis bag and dialyzed against PBS buffer overnight at 4°C with two changes during dialysis. The next day, analysis of the collected MBP-PE_PGRS45 protein and its concentration was determined based on SDS-PAGE analysis and Bradford assay, respectively. The purified MBP-PE_PGRS45 protein was estimated by densitometric analysis using ImageJ software.

### Factor Xa Digestion and Purification of MBP-PE_PGRS45 Protein

The purified MBP-PE_PGRS45 protein was dialyzed against factor Xa reaction buffer (50 mM Tris, 1 mM CaCl_2_, 0.1% Tween-20, pH 8.0) at 4°C overnight with two changes during dialysis. After dialysis, the MBP-PE_PGRS45 protein was incubated with factor Xa protease for 36 h at 4°C to release PE_PGRS45 protein from MBP-PE_PGRS45 protein. The above cleavage mixture was passed through a Ni-NTA agarose resin, then collect MBP removed MBP-PE_PGRS45 protein.

### Production and Identification of PE_PGRS45 Polyclonal Antibody

The immunization schedule used three New Zealand white SPF rabbits. Injections were subcutaneous (SQ) as emulsions in incomplete Freund's adjuvant (IFA). The adult male New Zealand white rabbits were first immunized subcutaneously with 400 μg PE_PGRS45 protein with an equal volume of IFA, and booster immunization was performed every 2 weeks for a total of 4 immunizations. Blood was collected 2 weeks after the end of immunization, and the titer of antiserum was determined by indirect ELISA method, after which blood was collected from the heart and the serum was separated. The main steps to collect blood from the heart included: 1) The New Zealand white rabbit was placed on its back and its limbs were tied to the animal cage. 2) The rabbit's fur on the left side of the chest was trimmed away and the skin was disinfected. 3) Pressing the left thumb against the xiphoid process of the sternum, the index and middle fingers were placed on the right chest, gently pushing the heart to the left to fix the heart on the left chest, and then the strongest part of the heart was touched with the left thumb. 4) Next, a 50 ml syringe (connected with a 16-gauge needle) tilted at a 45-degree angle was used to pierce the heart and draw blood from the strongest part of the heartbeat. 5) The blood drawn was immediately and poured into a sterile Erlenmeyer flask, and the serum was separated after coagulation. 6) The New Zealand white rabbits were sacrificed by injecting air into the ear vein. 7) The antibody production protocol was reviewed and approved by the Animal Ethics Committee of Bengbu Medical College (Approval No. 2020-127).

Antibody titer was measured using an indirect enzyme-linked immunosorbent assay (ELISA). The PE_PGRS45 protein (5 μg/ml) was coated on a 96-well microplate with 100 μl/well, overnight at 4°C. The coating solution was discarded and the plate was washed with PBS-Tween buffer (0.05% Tween 20 in PBS). The coated wells were blocked with 3% BSA for 1 h at 37°C. Then, the anti-PE_PGRS45 polyclonal antibody was diluted to 0.12 μg/ml, starting with 1:1000, then double-fold dilution, at 100 μl/well. This was incubated at 37°C for 1 h, and then incubated with added blocking solution, and blocked at 37°C for 1 h. The blocking solution was discarded and the plate was washed with PBST. Then, 1:5000 dilution of HRP-labeled goat anti-rabbit IgG (100 μl/well) was added and incubated at 37°C for 1 h. Finally, TMB coloring solution was added (100 μl/well), 37°C, protected from light for 15 min, and the A450 nm value was detected using a microplate reader.

## Results

### Sequence Analysis of PE_PGRS45

**Fig. 1:**
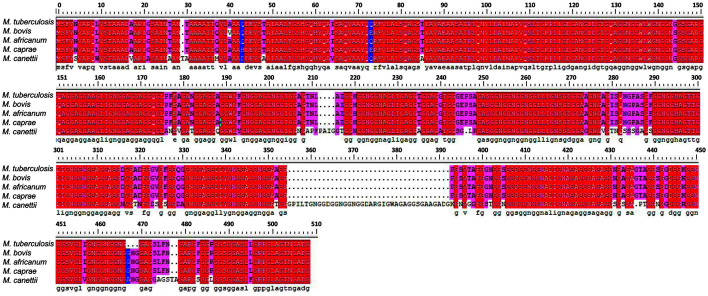
The multiple sequence alignment of PE_PGRS45 and its orthologs. Multiple sequence alignment between *M. tuberculosis* PE_PGRS45 and its homologs was performed using BLAST.

*M. tuberculosis* PE_PGRS45 gene has 1,386 bp and encodes a protein of 39.3 kDa. By running a BLAST search in the NCBI databases (http://blast.ncbi.nlm.nih.gov/), we found that PE_PGRS45 was highly conserved among pathogen mycobacteria (*M. Tuberculosis*, *M. bovis*, *M. africanum*, *M. caprae*, and *M. Canettii*) and was absent in non-pathogenic strains like M. smegmatis (Fig. 1). That implied PE_PGRS45 might be a virulence factor.

### Synthesis of the Codon-Optimized PE_PGRS45 Gene

**Fig. 2:**
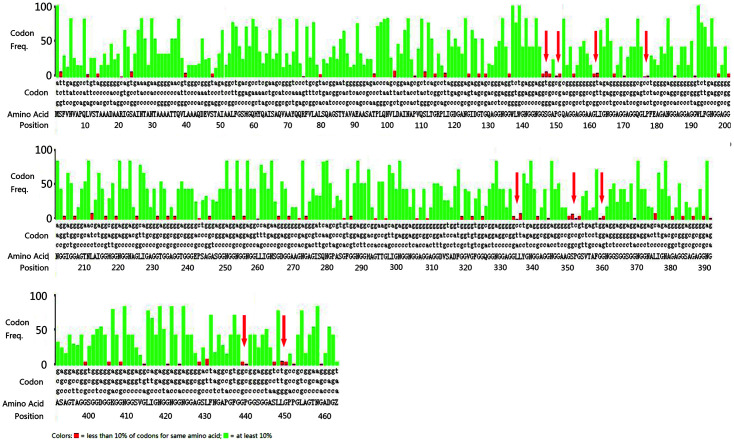
Analyses of codon usage in the original PE_PGRS45 gene encoding the PE_PGRS45 protein. The red bars present the rare codon with a frequency below 5% in *E. coli* and the red arrows present the tandem or triple repeats of rare codons.

The codon optimization of the gene sequence has a significant influence on the translation rate and overall protein production yield [15, 16]. Therefore, we investigated the codon usage of the PE_PGRS45 gene using *E. coli* codon usage analyzer 2.1 (http://www.faculty.ucr.edu/~mmaduro/codonusage/usage.htm). We found that the PE_PGRS45 gene has 20% rare codons with frequencies below 6% (Fig. 2). Several tandem/triple rare codons resulting in slow translation or early termination translation were also found, leading to a low protein yield. The codon adaptation index (CAI) for heterologous expression of original and optimized PE_PGRS45 nucleotide sequences in *E. coli* was calculated using an online rare codon analysis tool (http://www.genscript.com/cgi-bin/tools/rare_codon_analysis). For better expression of target proteins, the ideal range of CAI was found to be 0.8-1.0 [17]. The CAI of optimized PE_PGRS45 was observed to increase from 0.73 to 0.90. The PE_PGRS45 genés codons were optimized to *E. coli*-preferable codons for high-level expression.

### Construction of PE_PGRS45 Expression Plasmids

**Fig. 3:**
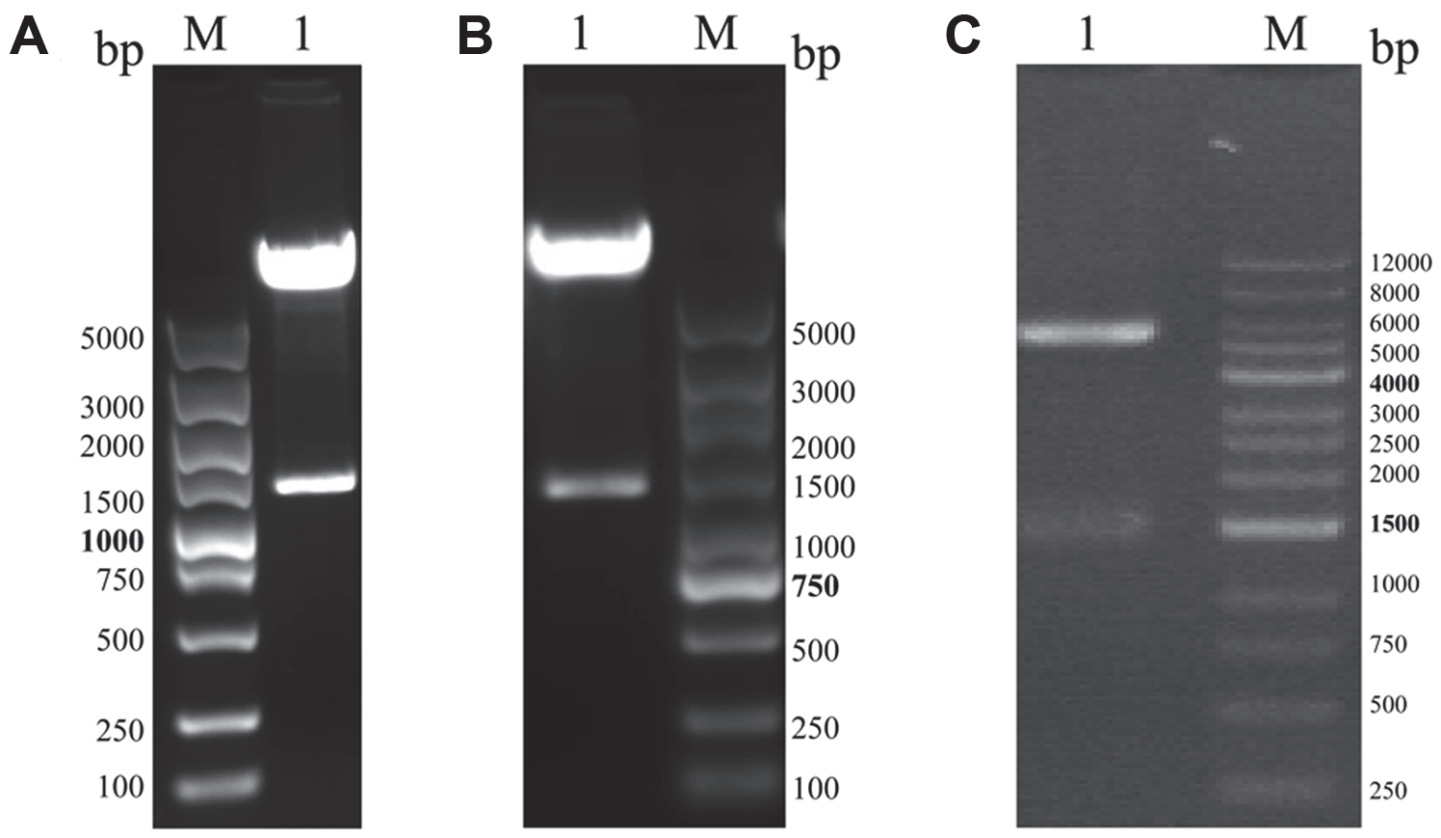
Identification of recombinant plasmid. (**A**) Plasmid pET-28a-PE_PGRS45 digested by NcoI/XhoI. Lane M: DNA marker; lane 1: plasmid pET-28a-PE_PGRS45 digested by NcoI/XhoI. (**B**) Plasmid pET-32a-PE_PGRS45 digested by NcoI/XhoI. Lane M: DNA marker; lane 1: plasmid pET-32a-PE_PGRS45 digested by NcoI/XhoI. (**C**) Plasmid pMAL-c5x- PE_PGRS45 digested by NdeI/HindIII. Lane M: DNA marker; lane 1: plasmid pMAL-c5x-PE_PGRS45 digested by NdeI/ HindIII.

An exogenous gene constructed in different expression vectors and expressed in different conditions might result in disparate expression levels and distinctly different protein stabilities for the target protein [18, 19]. We thus constructed three PE_PGRS45 recombinants with varying tags of fusion to screen the optimal recombinant for expressing the target protein. The PE_PGRS45 gene was then cloned into pET-28a, pET-32a, and pMAL-c5x vectors, respectively. The restriction analysis of pET-28a-PE_PGRS45 and pET-32a-PE_PGRS45 plasmids using NcoI and XhoI released the PE_PGRS45 insert of 1,386 bp (Figs. 3A, 3B), and pMAL-c5x-PE_PGRS45 plasmid using NdeI and HindIII individually showed bands at 1,386 bp (Fig. 3C), which confirmed successful cloning of the PE_PGRS45 gene into pET-28a, pET-32a, and pMAL-c5x vectors. The sequencing results complied with the codon-optimized sequence of the synthetic PE_PGRS45 gene and confirmed that the nucleotide sequence of the inserted fragments is correct.

### Small-Scale Expression and Solubility Testing

**Fig. 4:**
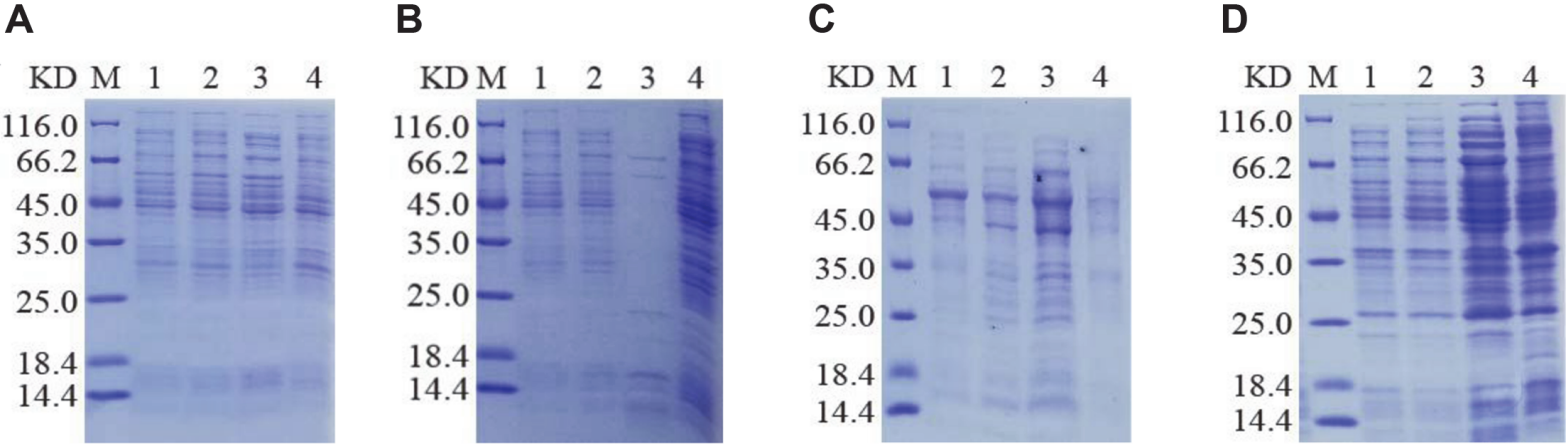
SDS-PAGE analysis of expression of PE_PGRS45 recombinant protein by pET-28a and pET-32a expression vectors after PE_PGRS45 codon optimization. Fusion expression of pET-28a-PE_PGRS45 in *E. coli* Arctic Express (DE3) at 20°C (**A**) and 37°C (**B**). Fusion expression of pET-32a-PE_PGRS45 in *E. coli* Arctic Express (DE3) at 20°C (**C**) and 37°C (**D**). Lane M, Protein marker; lane 1, *E. coli* cells before induction; lane 2, *E. coli* cells after induction; lane 3, supernatant of lysate; lane 4, precipitant of lysate.

**Fig. 5:**
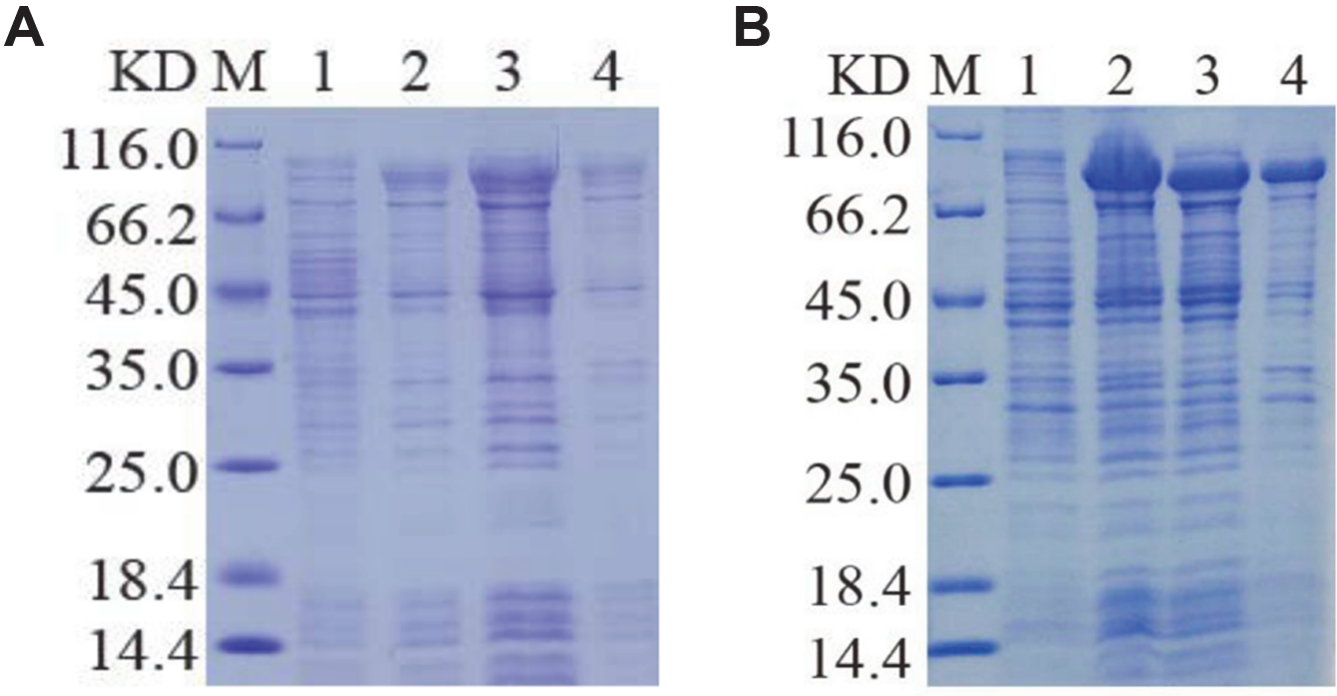
SDS-PAGE analysis of expression of PE_PGRS45 recombinant protein by pMAL-c5x expression vectors after PE_PGRS45 codon optimization. Fusion expression of pMAL-c5x-PE_PGRS45 in *E. coli* Arctic Express (DE3) at 20°C (**A**) and 37°C (**B**). Lane M, Protein marker; lane 1, *E. coli* cells before induction; lane 2, *E. coli* cells after induction; lane 3, supernatant of lysate; lane 4, precipitant of lysate.

The PE_PGRS45 recombinant protein was produced in *E. coli* Arctic Express (DE3) using the pET-28a, pET-32a, and pMAL-c5x vectors. We performed small-scale expressions to evaluate soluble protein productions in *E. coli* Arctic Express (DE3) host strains for the three prokaryotic expression plasmids with different fusion tags. The recombinant proteins were preliminary induction experiments with 0.5 mM IPTG at 20°C and 37°C for 12 h. The expression of pET-28a-PE_PGRS45 and pET-32a-PE_PGRS45 was examined by SDS-PAGE analysis. As shown in Figs. 4A-4D, the high induction temperature (37°C) and lower temperature (20°C), showed that no obvious new bands were observed in the supernatant and precipitate of cell lysate. Then, the recombinant plasmid pMAL-c5x-PE_PGRS45 was transformed into *E. coli* Arctic Express (DE3). Without induction, MBP-PE_PGRS45 showed no observed fractions as indicated in Fig. 5A of lanes 1 and Fig. 5B of lanes 1, respectively. Following induction with IPTG, an intense band was observed in supernatant extract (Fig. 5A, lanes 2 and Fig. 5B, lanes 2), indicating that the overexpressed MBP-PE_PGRS45 was mainly expressed in the supernatant extract (Fig. 5A, lanes 3 and Fig. 5B, lanes 3). In addition, lower induction temperatures (20°C) caused a marked decrease in MBP-PE_PGRS45 expression. The molecular mass of the expressed protein in the bacterial lysis extract was approximately 82 kDa.

### Purification of Recombinant MBP-PE_PGRS45 Protein

**Fig. 6:**
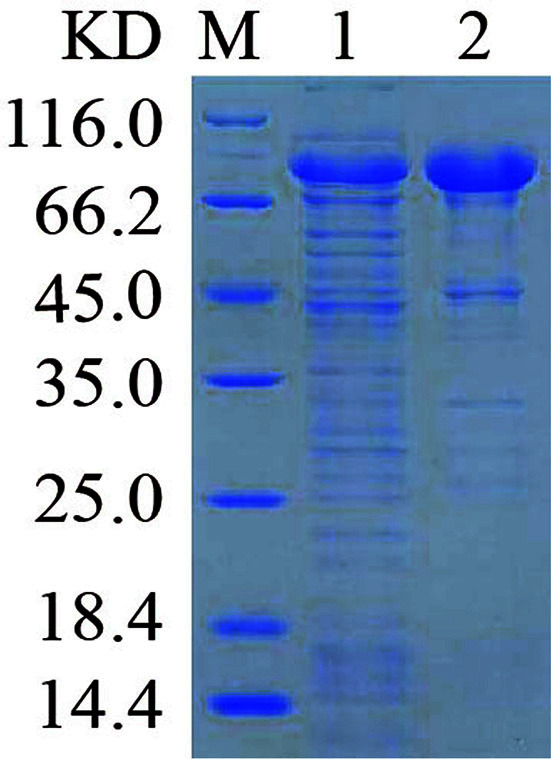
SDS-PAGE analysis of the purified recombinant PE_PGRS45 protein. Lane M, Protein marker; lane 1, Flow fluid; lane 2, Purified PE_PGRS45.

The MBP fusion construct shows high PE_PGRS45 expression levels and solubility. The filtered supernatant was accomplished by immobilized metal affinity chromatography (IMAC) on a Ni-NTA resin column to obtain MBP-PE_PGRS45. The final purity was estimated to be greater than 90% by optic densitometry of the SDS-PAGE gels (Fig. 6).

### Separation of the PE_PGRS45 Proteins from the MBP Fusion Proteins by Factor Xa Digestion

**Fig. 7:**
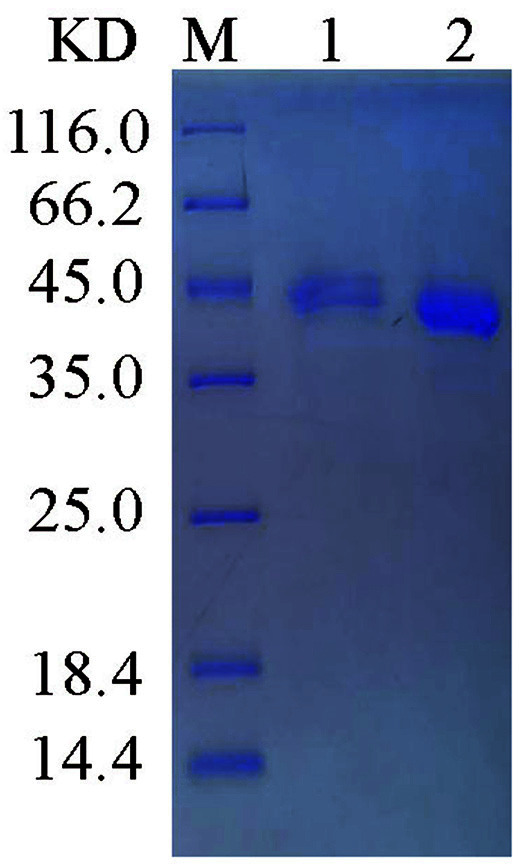
SDS-PAGE analysis of factor-Xa cleaved MBP-PE_PGRS45. Lane M, Protein marker; lane 1, MBP tag (43 kDa) cleaved using the factor-Xa; lane 2, Final purified PE_PGRS45 (39 kDa).

The pMAL-c5x expression vector contained factor Xa cleavage site (Ile-Glu-Gly-Arg) between the fusion partner (MBP) and the target proteins. To examine whether the MBP fusion protein could be cleaved off from the MBP-PE_PGRS45 proteins, purified MBP-PE_PGRS45 proteins were incubated with factor Xa. SDS-PAGE analysis of the factor Xa digestion showed a complete disappearance of the 82 kDa MBP-PE_PGRS45 proteins after 36 h of incubation along with an appearance of two major bands of around 43 kDa and 39 kDa (Fig. 7), indicating a cleavage of rMBP-PE_PGRS45 proteins into MBP (43 kDa) and PE_PGRS45 proteins (39 kDa).

### Antiserum Titer Determination by ELISA

**Fig. 8:**
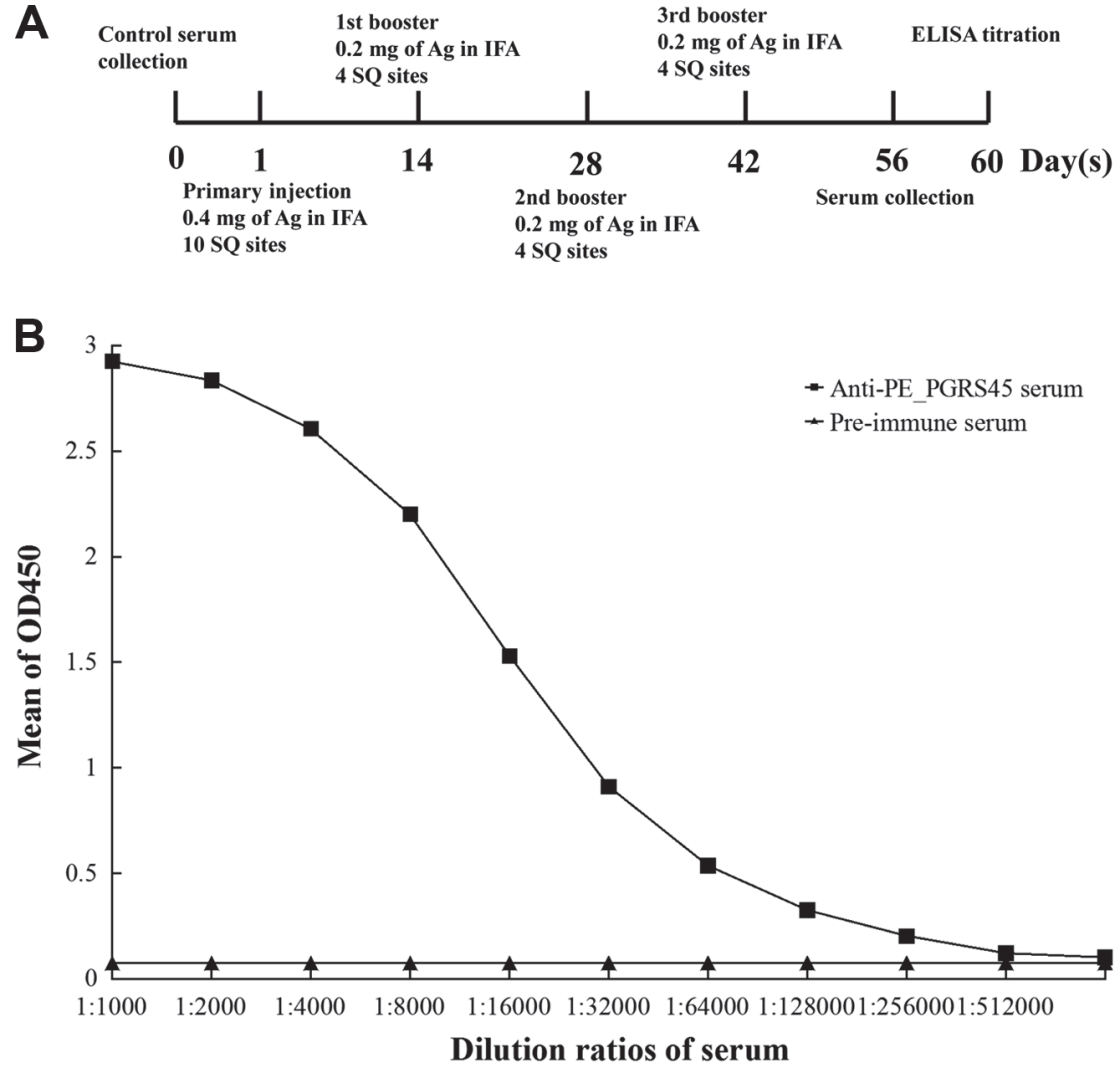
The process of rabbit immunization (**A**) and indirect ELISA determined titer of anti-PE_PGRS45 polyclonal antibody (**B**).

After the rabbits were immunized four times with the purified PE_PGRS45 (Fig. 8A), the antisera were tested at different dilutions from 1:1000 to 1:1024000 by ELISA. As shown in Fig. 8B, the absorbance value ratio of antiserum to negative serum is 2.27 when the ratio is 1:256000. Therefore, the maximum titer of the antiserum was determined to be 1:256000.

## Discussion

*pe*/*ppe* genes are selectively expressed under stress conditions or during infection, suggesting roles in mycobacterial pathogenesis [20]. Studies have demonstrated that PE_PGRS45 was constitutively expressed under various environmental conditions (such as nutrient depletion, hypoxia, and low pH) of the in vitro growth conditions examined, indicating that PE_PGRS45 protein is critical to the essential functions of *M. tuberculosis* [21]. Srivastava *et al*. found that the expression of PE_PGRS45 gene was significantly upregulated after *M. tuberculosis* acted on macrophages in vitro or the lungs of mice infection [22]. This implies that PE_PGRS45 may play a role in *M. tuberculosis* infection and pathogenesis, but the mechanism is unknown.

PE_PGRS45 is a 461-amino acid protein that shows a very high similarity with the N-terminal domain of PE_PGRS17 and PE_PGRS18. However, a substantial variation is observed between the amino acid sequences in the PGRS domain [13]. Corresponding verification was carried out with our prepared PE_PGRS45 antibody, and some interesting results were also obtained, but further verification is needed (Figs. S1-S3). Strong M *et al*. attempted to individually express PE_PGRS45, but it was not expressed in *E. coli* [23]. The fact that the gene of *M. tuberculosis* is not easily expressed in *E. coli* may be mainly due to the high content of G + C and the use of unique codons [24]. Fusion tags are indispensable tools to improve recombinant protein soluble expression and accelerate the characterization of protein structure and function [25]. His-Tag is used to aid dissolution and folding under native or denaturing purification conditions [26]. Thioredoxin (Trx) can reduce disulfide bonds in proteins, promoting correct folding of the amino acid chain and preventing the generation of inclusion bodies [27]. MBP acts as a general molecular chaperone that promotes proper folding into native conformation, and has the highest ability to improve soluble levels [28]. MBP is also resistant to proteolysis and may protect its partner protein from degradation [29]. It is characterized by improving the expression level and solubility of the expressed product [30]. pMAL expression vector system was designed to express His6-MBP fusion protein to facilitate protein folding and affinity purification [31]. However, little is known about the specific function of PE_PGRS45 due to the difficulty in preparing the high-quality protein.

In the present study, His6, Trx, His6-MBP were used as fusion tags, but only MBP-PE_PGRS45 formed soluble expression. The purification using His6-MBP tag-specific binding to the Ni column is also easy to separate after the tag cleavage. Removal of the MBP fusion protein by factor-Xa cleavage did not alter the solubility of PE_PGRS45, supporting that PE_PGRS45 was properly folded and fusion protein with good purity was obtained. We used the purified PE_PGRS45 protein to immunize New Zealand rabbits and got anti-PE_PGRS45 serum. The titer was found to be higher than 1:256000. This shows that the purified PE_PGRS45 protein can induce New Zealand rabbits to produce high-titer antibodies. Therefore, the results of this study lay the basis for the further resolution of the crystal structure of PE_PGRS45, as well as the study of potential function. In addition, this will be followed by further evaluation of these specific antigens to develop highly sensitive and specific diagnostic tests for tuberculosis.

## Supplemental Materials

Supplementary data for this paper are available on-line only at http://jmb.or.kr.

## Figures and Tables

**Table 1:** Primers used for the construction of recombinant plasmids.

Expression vector	Primer name	Primer sequence (5′-3′)^a^	Restriction site
pET28a	28aFP	CATGCCATGGATGAGCTTTGTGAATGTGGCC	Nco I
	28aRP	CCGCTCGAGTTAACCATCGGCACCGTTGGTACC	Xho I
pET32a	32aFP	CATGCCATGGATGAGCTTTGTGAATGTGGCC	Nco I
	32aRP	CCGCTCGAGTTAACCATCGGCACCGTTGGTACC	Xho I
pMAL-c5x	c5xFP	GGAATTCATATGAGCTTTGTGAATGTGGCC	Nde I
	c5xRP	CCCAAGCTTTTAACCATCGGCACCGTTGGTACC	Hind III

^a^The single underlined sequences indicate restriction enzyme sites.
