# Supplementary material for: Codon Optimization, Soluble Expression and Purification of PE_PGRS45 Gene from Mycobacterium tuberculosis and Preparation of Its Polyclonal Antibody Protein
Source: J Microbiol Biotechnol. 2021 Sep 1;31(11):1583–90. doi: 10.4014/jmb.2106.06006 (PMC9705950; doi:10.4014/jmb.2106.06006)
Supplement: Supplementary file 1 [file jmb-31-11-1583-supple.pdf]

J. Microbiol. Biotechnol.
https://doi.org/10.4014/jmb.2106.06006

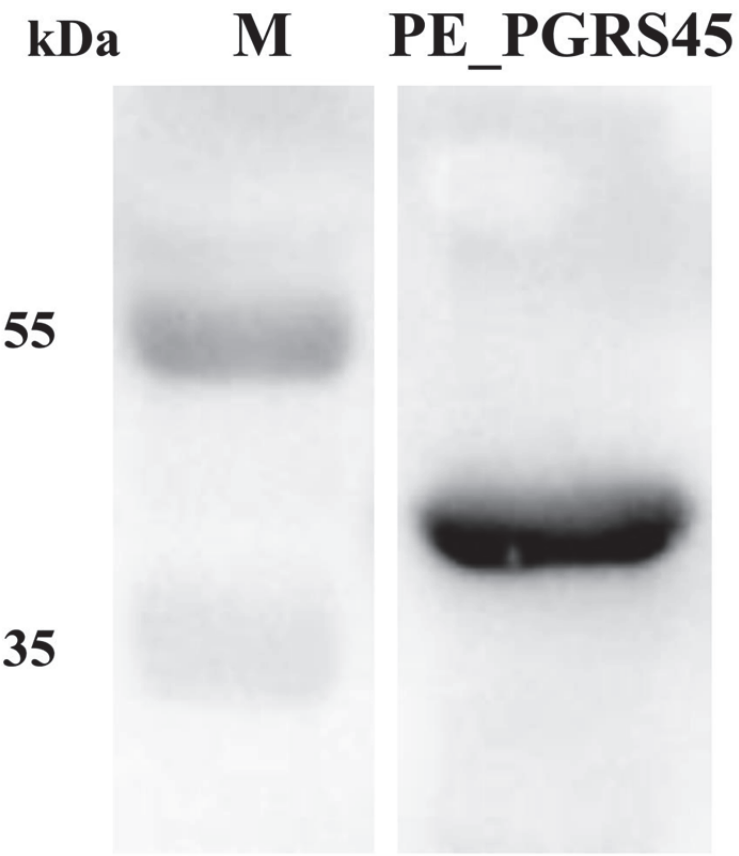

Supplementary Figure 1 Western Blot detection specificity of polyclonal antibody to PE_PGRS45recombinant protein.

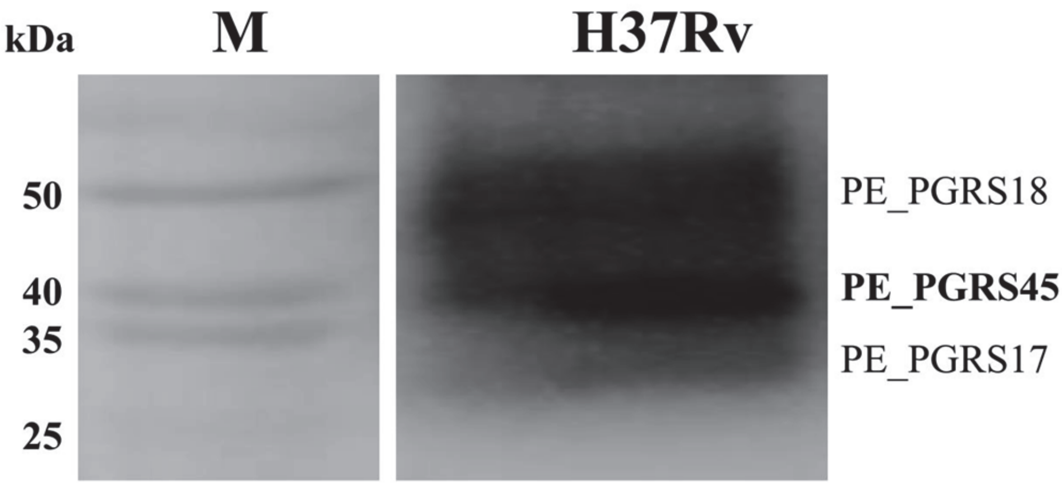

Supplementary Figure 2 Western Blot detection specificity of PE_PGRS45 polyclonal antibody toM. tumerculosis.

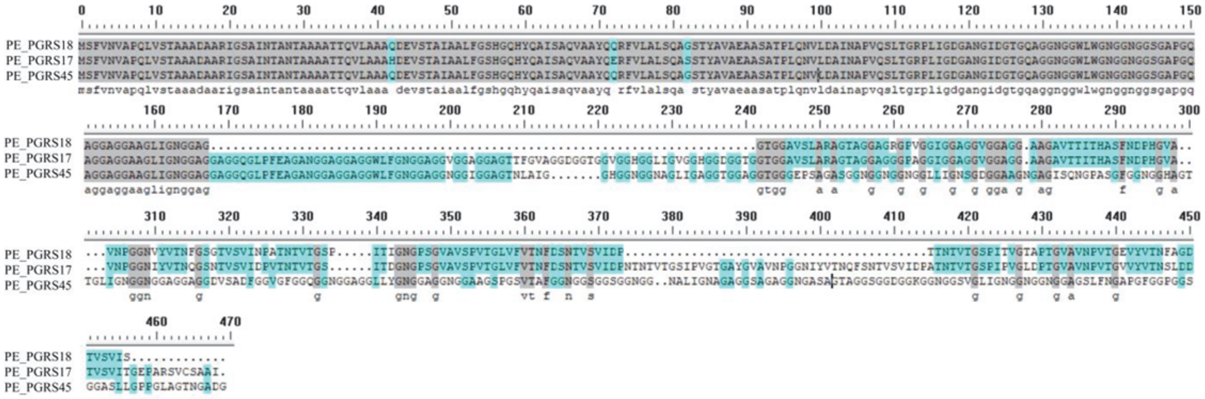

Supplementary Figure 3 Sequence alignment analysis ofM. tuberculosisproteins PE_PGRS17, PE_PGRS18 and PE_PGRS45.
